# PCRdrive: the largest qPCR assay archive to date and endless potential for lab workflow revitalization

**DOI:** 10.1186/s12859-018-2452-3

**Published:** 2018-11-22

**Authors:** Florian Burger, Michele Angioni, Gianluca Russo, Martina Schad, Jim Kallarackal

**Affiliations:** OakLabs GmbH, Neuendorfstr. 16B, Hennigsdorf, 16761 Germany

**Keywords:** RT-qPCR assay, SNP check, Specificity check, Electronic lab notebook, Online workspace, Assay database

## Abstract

**Background:**

Primer design is a crucial step in establishing specific and sensitive qPCR assays. Even though numerous tools for primer design exist, the majority of resulting assays still requires extensive testing and optimisation or does not allow for high quality target amplification. We developed a workflow for designing qPCR assays. Unlike other tools, we compute a PCR assay including primer design, concentrations and the optimal PCR program.

**Results:**

Gene expression assays were already generated in a total of 283,226 genes from three species and are continued for all genes of the major model species. The results are available online at https://pcrdrive.com/lab#/assay-database. The workflow involves filtering Primer3-generated primers by considering diverse parameters including specificity, single-nucleotide polymorphisms (SNPs), secondary structure as well as compatibility with standard qPCR assay conditions. The resulting assays consist of transcript-specific primer sequences, a reagents protocol as well as instrument settings which are provided in a web-based tool called PCRdrive. PCRdrive was designed to support PCR users in their PCR-related tasks and is equipped with handy functions, components of an electronic lab notebook (ELN) as well as teamworking opportunities.

**Conclusion:**

High quality ready to use qPCR assays for gene expression analysis are provided within the online platform PCRdrive. A built-in primer designer enables easy generation of assays which is not supported by any other tool. The wet lab optimisation of new assays can be transparently documented and shared within the team. PCRdrive also contains an archive of public PCRs which is updated regularly. Users may use the archive to publish their PCR to the community which makes it easy for other researchers worldwide to reproduce and validate the PCR. PCRdrive is a growing network of PCR users, simplifying and streamlining research through its useful existing features and continuous developments from the active development team.

## Background

Real-time PCR is a widely used sensitive and reliable method to study gene expression levels of a limited number of genes. It is used in many fields such as biomedical research and diagnostic testing as well as verification of microarray and RNA seq data.

Establishing stable and sensitive assays involves designing good pairs of primers which is by no means a straight forward task. Primers should have a balanced G/C content, similar melting temperatures and no preference for self-complementarity or hair pin structure. Furthermore, SNPs should not lie within primer regions and the primer pair should only amplify the target of interest but not any unintended targets. In addition, usually the pairs’ absolute and relative binding positions to the target sequence have to fulfill constraints. Attention has to be paid to each of these factors as every step is crucial and might impede yielding a sensitive and specific assay [1]. Furthermore, the primers need to harmonise with universal reaction conditions in terms of the “mastermix” as well as the time and temperature profile of the reaction. Many public software tools are available to design PCR primers. The widely used Primer3 algorithm [2, 3] designs primers based on diverse parameters including some sort of secondary structure check. However, Primer3 neither performs a specificity analysis nor an SNP check. Alternatively, other tools can be used which rely on the Primer3 algorithm to generate primers and enrich the process with extra functions, such as specificity check (e.g. provided by Primer-Blast [4]), SNP check, exon-intron boundary etc.

To facilitate the process of establishing new qPCR assays, instead of designing primers researchers may use commercially available, partially wet lab validated primers or freely available primers in primer databases like PrimerBank [5], qPrimerDepot [6], GETPrime [7, 8], qPrimerDB [9]. Even single species primer databases exist, e. g. AtRTPrimer [10] (A. thaliana). Nonetheless, independently of the primers’ origin, the performance of each new PCR assay should be wet lab validated by researchers. PCR reagents, conditions and cycler settings should be documented to sustainably retain a stable assay within the research group. While researchers preferentially use paper lab notebooks (PLNs) due to familiarity and ease of use, ELNs enable unlimited comprehension and exchange of content which builds the basis for efficient and sustainable research.

In this work we have generated sets of high quality qPCR assays that perform reliably within universal experimental conditions that are embedded within an easy-to-use web platform that offers further useful features.

## Implementation

### PCRdrive

#### Database

The user data are stored in a SQL database, updated to the last available version. The table structure is rather involved as it has to reflect the many functionalities the application provides. It has been conceived to maximise both performance and reliability of stored information. Complex though highly performant queries are applied to retrieve the needed information.

#### Security

At PCRdrive, the user can rely on the fact that our strong security measures are deeply rooted in our highly reliable technical background. The entire website is SSL protected and communication with the servers are fully encrypted. User passwords are stored hashed relying on widely used and well accepted cryptography algorithms. Our databases are included in our emergency and disaster recovery strategies and data loss is prevented by backups on daily basis. All in all, we guarantee the highest level of user data privacy, backed by our use of a Europe based server hosting service. Under no circumstances will user data be sold or given to third parties. PCRdrive fully complies with th european general data protection regulation (GDPR).

#### Development

PCRdrive is being developed with a high level of professionalism, including diligent compliance with accepted programming and industry standards from the early development stages. An agile development process is used to continually improve the application, adding new features while maintaining the current code base. In the event of site complications, our active developers can address the problem, instantaneously fixing bugs thanks to real-time application error reporting. The most advanced technologies used in the back- and front-end development guarantee superior robustness and speed. Lastly, we are constantly improving the user experience by tracking activities as well as regularly gathering user feedback. Migrations and updates are managed by the renowned Ansible framework which is also used to manage large server farms. The complete code base and history is managed with the git version control system.

#### Support

Full support is guaranteed to the users: not only should the PCRdrive community receive regular updates - users can participate in the process by requesting features or even reporting bugs directly to the development team with real-time response. It is in this way that we create a true dialogue between the creators and the users of PCRdrive, to create the best and most productive experience for everyone involved.

### PCR assay generation

**Fig. 1:**
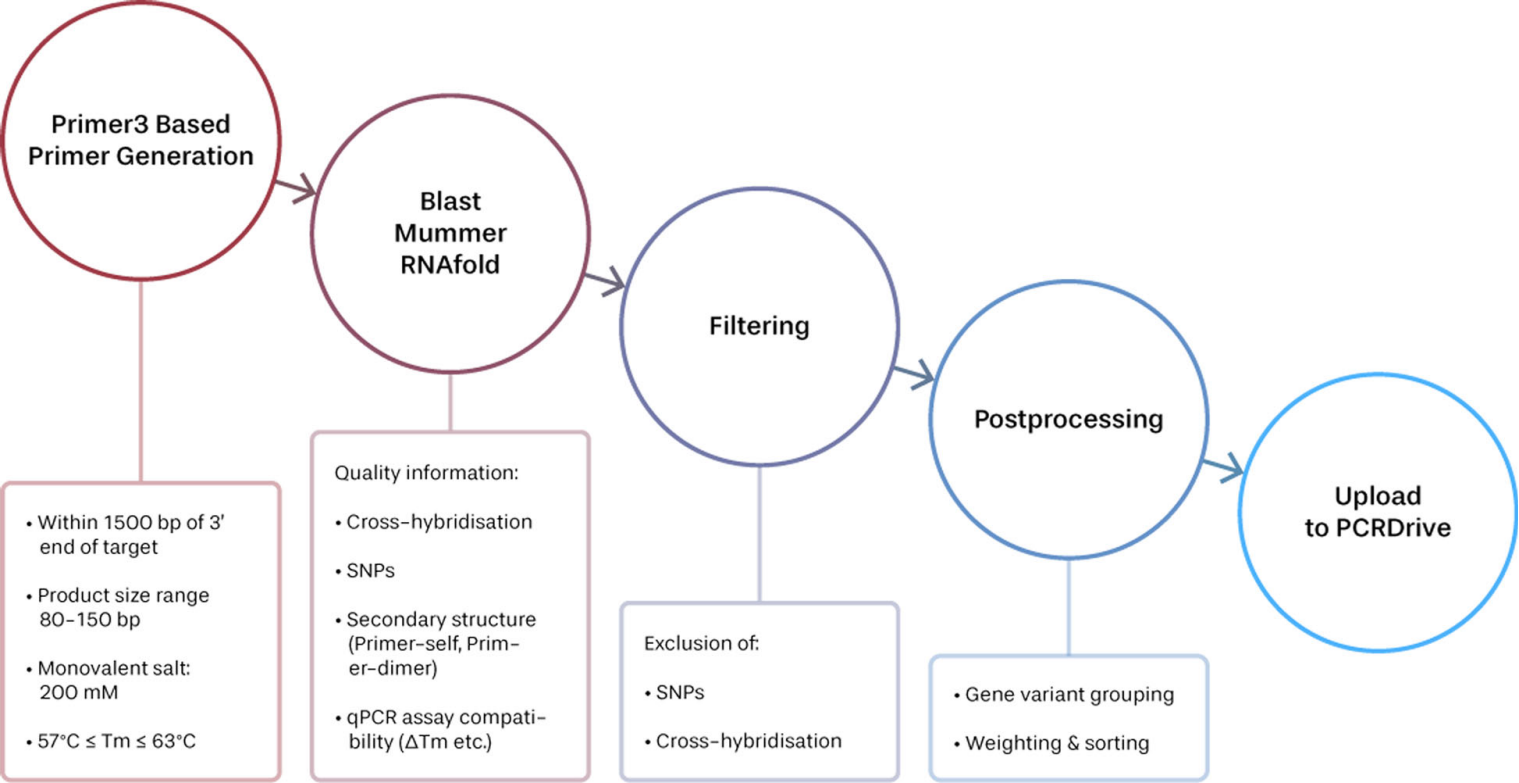
Schematic view of the workflow used for PCR assay generation. See text for details concerning each step involved

The workflow we have followed for generating the assays is summarised in Fig. 1 and each individual step is detailed in a separate paragraph. We start by generating a set of 200 primer pairs (c. f. “Primer generation” section) for each input gene target sequence and subsequently assessing the quality of each pair by computing several quality criteria measures (c. f. “Cross-hybridisations, SNP contamination, Secondary structure” sections). In filter and post-processing steps, we then restrict to primer pairs meeting our quality criteria and group them according to their sensitivity to different splice variants of the gene (c. f. “Categorisation and selection of primers” section) and choose the best primer pair for each gene variant category that is found. The PCR assays found in this way are then uploaded to PCRdrive.

#### Considered species

We have completed the full analysis, as described here, for three of the main model species, *Homo sapiens*, *Mus musculus* and *Rattus norvegicus*. However, several more species will follow (as indicated in the outlook, “Conclusion” section) in the near future. If a species is not (yet) present in our database, we encourage the user to request this species by providing web URLs to the resources needed to compute the assays. Alternatively, users can work in the meantime using PCRdrive’s built-in PurePrimer algorithm instead.

#### Primer generation

For generation of primers, we use Primer3’s *primer3_core* with default parameters. We deviate from the program’s defaults only for the parameters controlling the product size range, which we set to 80-150 bp, and for the optimal product size, which we set to 115 bp. For short input target sequences, primer3 core was not able to generate the requested number of primer pairs; in which case, we have reduced the input product size range to 50-70 bp and used an optimal product size of 60. Target sequences with lengthes below 50 bp have been excluded from analysis. The monovalent salt concentration is set to 200 mM, which has been found to better reproduce the experimentally measured primer melting temperature for the master mix we employ. Additionally, we rely on the salt correction by Owczarzy [11] for computing the primer melting temperature *T*_*m*_.

#### Cross-hybridisations

For checking the primers for potential cross-hybridisations to other gene targets within the same species, we use the *blast* program [12]. The database against which the similarity search is run has been generated from the same species’ transcriptome. Since smaller seed sizes than 6 exact bp matches were prohibitively costly in terms of computation time, we have used this number throughout our assays analysis. We used the *blast* version, optimised for short sequences, with an e-Value of 10 and otherwise relying on standard parameters for the searches. We consider every match with more than 0.7 similarity for both forward and reverse primers (using the primer length as reference) as a possible cross-hybridisation target. If furthermore, the matches lie on opposite strands and give rise to an amplicon of reasonable size (less than 20 times the targeted amplicon’s size) we store the information for further analysis.

There can now arise three possibilities concerning the set of found matching targets. First, a true cross-hybridisation due to matching with a highly different gene might occur, in which case the primer pair is to be discarded. Secondly, the primer pair might, in addition to the current gene of interest, cross-hybridise to one or more splicing variants of the gene (transcript variants). While the latter is the most frequent situation since no restriction on primer location concerning exon-intron boundaries has been imposed during primer generation, there also exists the situation that all gene variants are possibly amplified. As a third possibility, also a mixture of above two cases can arise at the same time, which again renders the primer pair unspecific.

#### SNP contamination

Each produced primer is checked for SNPs occurring within the primer’s binding region within the genome. We employ an exact sequence matching analysis that is conducted as detailed in the following description. Using the available genomic coordinate information (as provided by the NCBI prepared.gff file), we obtain the relevant coordinate regions within the genome for each considered gene of interest. Within these coordinate regions, we look up all contained SNPs employing the assembled SNP information (from.vcf files provided by NCBI) which is used together with the genome sequence to build up short genomic sequences surrounding the SNP sites symmetrically. We use 26 bases in both strand directions to build the corona around the permutation locations. We then produce exact alignments using *mummer* [13] between the primers and the prepared corona sequences which are used as the subject. We filter the apparent matches according to their length and location within the corona sequence and store the relevant matches with any related information (such as SNP id etc.) to mark the primer as possibly afflicted by the SNP.

#### Secondary structure

We perform secondary structure analysis for testing potentially harmful secondary structure possibly hampering the performance of the PCR. Single primer secondary structure is checked to detect possible hairpin structure of forward and reverse primers. Furthermore, we check for primer dimer structure of forward and reverse primer. For computation of secondary structure we use the ViennaRNA package [14]. Primer self-structure is tested with *RNAfold* and primer dimers are tested using *RNAcofold*. In all cases, we used the DNA parameter sets ’dna_mathews2004.par’ included in the package at a temperature of 60^∘^C, which reflects our choice of annealing temperature. The resultant information about the minimum free energy (MFE) and the related structure is stored for further post-processing and filtering.

#### Categorisation and selection of primers

The following quality criteria are imposed on the primer pairs, having passed the analysis steps described in above paragraphs A primer (either forward or reverse):

- must not have cross-hybridisations to other gene(s) other than the input gene of interest
- must not contain SNP(s)
- must have passed the criteria imposed at the primer generation step

If any of these criteria are violated by a primer, the corresponding primer pair is dropped and disregarded in the subsequent ranking. All primer pairs that passed up to now are sorted into categories reflecting the subset of gene splice variants that the pair is able to amplify. Depending on the number of existing (known) variants of the gene and on the similarities between the variants, several categories per gene may be realised by the found primers. The variants that are among the amplified products are made transparent in the description section of the PCR assays. Within each of the found gene variant categories, the primer pairs are then sorted by the MFE of a possible primer pair dimer structure and we use the one with the lowest probability to build the dimer as our final result.

#### Genome and SNP data source

For retrieval of whole transcriptome sequences and related genome information as well as the SNP database we rely on the NCBI FTP download service.

For *Homo sapiens* we used the download links ftp://ftp.ncbi.nlm.nih.gov/genomes/all/GCF/000/001/405/GCF_000001405.36_GRCh38.p10/ as well as ftp://ftp.ncbi.nlm.nih.gov/snp/organisms/human_9606_b151_GRCh38p7/VCF/, of which we only considered SNPs with alternative allele realisation frequencies of at least 1% in the analysis.

For *Mus musculus*, the exact links are ftp://ftp.ncbi.nlm.nih.gov/genomes/all/GCF/000/001/635/GCF_000001635.25_GRCm38.p5 for the genome and transcriptome information and ftp://ftp.ncbi.nlm.nih.gov/snp/organisms/archive/mouse_10090/VCF/ for the SNP information. For *Rattus norvegicus*, the link references are ftp://ftp.ncbi.nlm.nih.gov/genomes/all/GCF/000/001/895/GCF_000001895.5_Rnor_6.0 and ftp://ftp.ncbi.nlm.nih.gov/snp/organisms/archive/rat_10116/VCF/. Note that the Y-chromosome SNP information is missing in the data source for this case. We have checked for each species that the available SNP information is consistent with the genome information by checking for each SNP record that the indicated reference allele is matched by the genome at the indicated locus.

#### Implementation details

For the generation of the PCR assays, a *python* package has been written that provides common interfaces to the below mentioned sequence analysis software packages. Apart from primer generation, *primer3_core* does not provide native parallel execution support but is nonetheless fast enough to not create a bottleneck for the computation, all steps in our workflow have been parallelised in order to take advantage of today’s multi-processor machines and multi-core CPUs. In this way, it was possible to reduce the necessary per assay wall clock time significantly. The per species accumulated computational cost measured in hours per CPU core is displayed in the last column of Table 1.

**Table 1:** PCR Assay summary

Species	*N*_T_	*N*_A_	*N*_E_	Cost [core-h]
*Homo sapiens*	153,978	162,512	34,683 (661)	50,513
*Mus musculus*	107,188	194,305	6,120 (332)	8069
*Rattus norvegicus*	69,440	109,051	5,473 (111)	4352

For each species, we show the number of considered input targets *N*_T_ and the total number of generated PCR assays *N*_A_, which comprises all found gene variant categories. The numbers of failed and therfore excluded assays is shown in the column denoted by *N*_E_. While the first number we show is the number of assays for which no primer pair fulfilling our quality criteria could be found among the 200 primer pairs considered, the second number represents the assays for which no primers could be found due to exceedingly short sequence length. The last column indicates the total amount of computation time spent in units of core-hours to generate the complete set of PCR assays

## Results

### PCR assay results

#### Assay statistics

For our sets of PCR assays we have gathered statistics related to the number of primer pairs that:

- have been considered in the analysis *n*_p_,
- have been discarded due to SNP contamination *n*_SNP_
- have been discarded due to cross hybridisation with other genes *n*_X_

The numbers are gathered in Table 2.

**Table 2:** Primer statistics

Species	*n*_p_	*n*_X_	*n*_SNP_
*Homo sapiens*	30,663,400	4,886,495	26,572,208
*Mus musculus*	21,371,200	2,642,881	10,464,566
*Rattus norvegicus*	13,865,800	2,214,656	684,033

For each species we show the total number of considered primer pairs, the number of pairs that potentially create cross-hybridisations as well as the number of pairs contaminated with SNP(s)

**Fig. 2:**
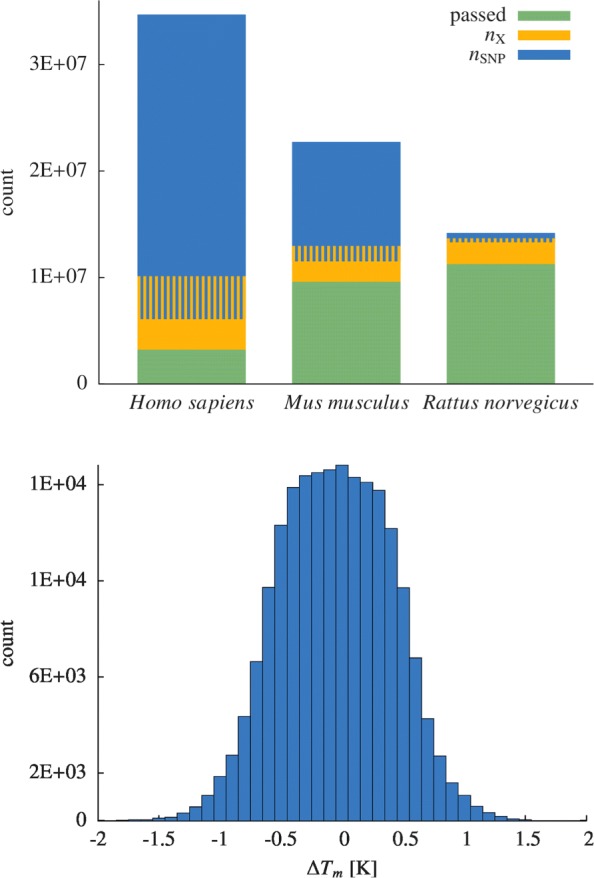
Discarded primer pairs due to a contained SNP and due to unintentded cross-hybridisation to other genes relative to the generated number of primers for *Homo sapiens*, *Mus musculus* and *Rattus norvegicus* (top). Histogram of the difference in melting temperature of forward and reverse primers *ΔT*_*m*_ for the generated PCR assays for *Mus musculus* (bottom)

**Fig. 3:**
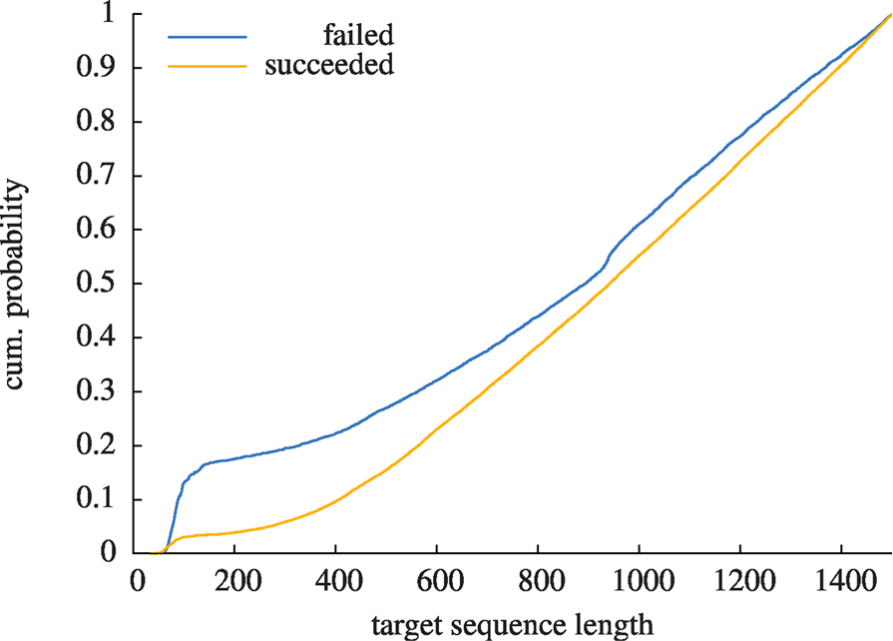
Cumulative probability of failed and successfully generated pcr assays depending on input target gene sequence length. The case of *Homo sapiens* is shown

We have visualized the impact of the two main quality restrictions, SNPs and cross-hybridisations, in Fig. 2 where we show the amount of primer pairs that needed to be discarded due to either of the two. It can be seen that a large fraction of generated primers would indeed be unspecific and possibly lead to amplification of unwanted genes or would perform unreliably as their binding sites include SNP mutations. While a significant fraction of the generated primer pairs would lead to unspecific amplification we find that the dominant reason of discarding primer pairs for *Mus musculus* being contained SNP sites in either of the two primer sequences. For *Rattus norvegicus* we find the opposite as more primer pairs were discarded due to cross-hybridisation than due to SNPs. The difference in number of found SNP contaminated primer pairs between the two species is striking; this can be attributed to the fact that the total number of reported SNPs in the NCBI SNP database for *Mus musculus* is more than ten times larger than the number of SNPs for *Rattus norvegicus*. For *Homo sapiens*, for which the SNP database is even larger than for *Mus musculus*, finding valid primer pairs becomes even more difficult as can be seen from the left most bar of Fig. 2. We find the most extreme case there, with a large fraction of primer pairs filtered out due to SNP contamination. In order to analyse the situation more closely for *Homo sapiens* we have compared the lengthes of target gene sequences of failed assays (i.e. for which all primer pairs had to be discarded) with the sequence lengthes of genes for which at least one assay could be generated successfully. Since our primer generation strategy imposes a restriction of 1500 base pairs from the 3^′^ end of the input sequence we have only studied targets whose sequence length was within this bound. The outcome of this analysis is shown in Fig. 3 where the cumulative distribution function (the cumulative probability) for the two cases is shown. As can be seen from the fact that the curve for the failed assays rises earlier than the curve for successful assays it seems more difficult for our primer generation algorithm to find SNP-free primers for small input target sequences, which matches the naive expectation one could have.

**Fig. 4:**
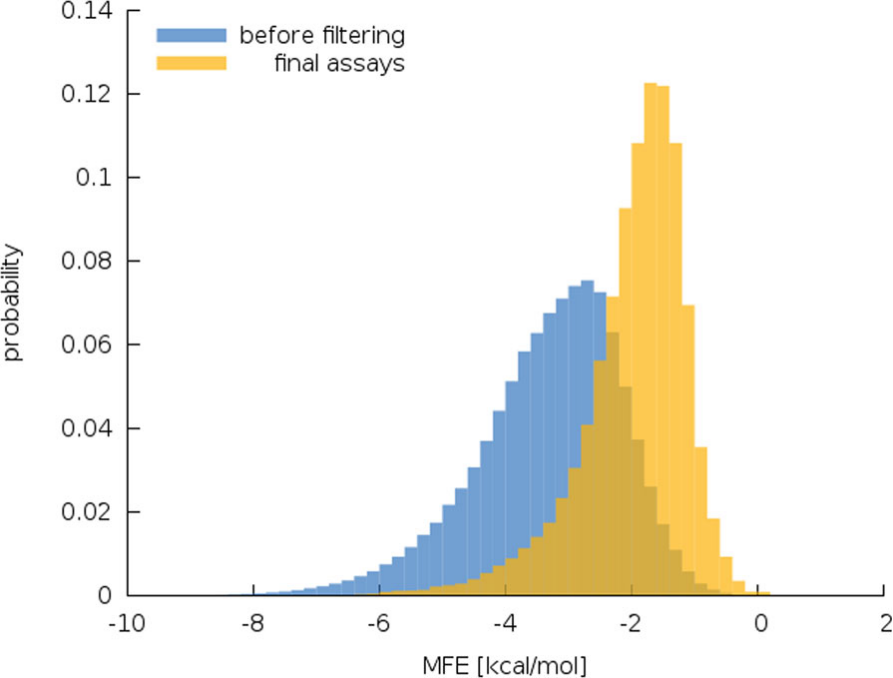
Distribution of primer pair secondary structure dimer MFE before filtering and of our generated assays, i.e. after sorting for largest MFE and choosing the primer pair having highest MFE. The case of *Mus musculus* is shown

The effect of sorting the primer pairs that pass the filtering by the MFE associated to generating a dimer secondary structure is shown in Fig. 4 where we show the normalised probability distribution of found dimer MFE once without filtering and once including only the selected primer pair(s) of the generated PCR assays for the case of *Mus musculus*. As a consequence of our strategy of sorting the primer pairs by their MFE and choosing the one with largest value our generated PCR assays will have reduced probability of showing primer dimer amplification. Furthermore, also the tail of the distribution towards large negative MFE is suppressed which should manifest itself in lower risk of overall failure of PCR due to preferred binding of primers to each other instead to their according template sequence. While the lowest MFE found before the sorting and filtering procedure occurs at −15.6 kcal/mol for our final assays we find the minimum at −11.4 kcal/mol.

#### Transcript coverage

**Fig. 5:**
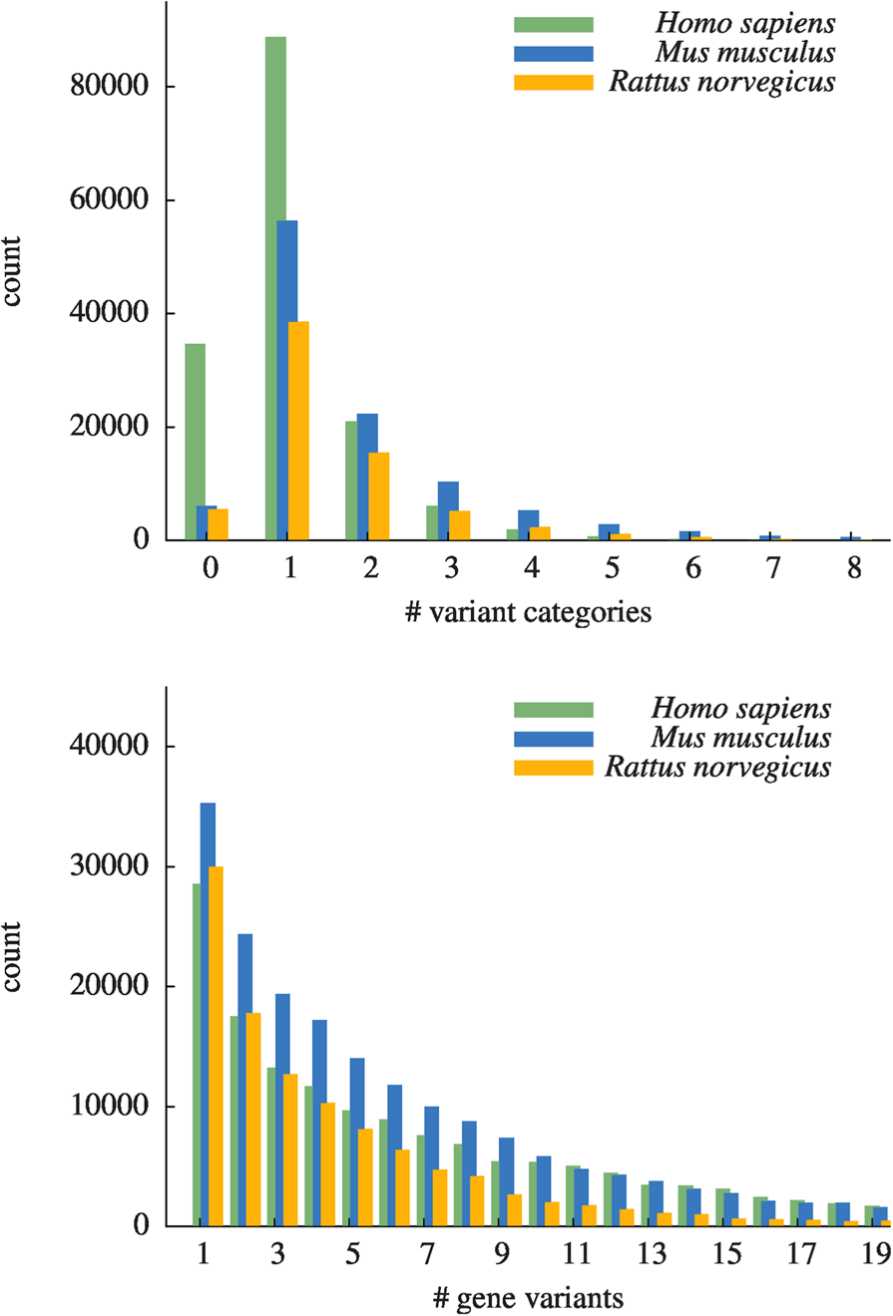
Distribution histogram for number of generated transcript variant categories for our assays (top). Distribution histogram of per category amplified gene variants (bottom)

As explained above we store each primer pair amplifying a different subset of a given input target gene’s splice variants as a seperate PCR assay. In Fig. 5 (top) we show a histogram of found PCR assays per gene. As can be seen we mostly find a single PCR assay per gene, however a significant number of genes produces more than a single gene variant category and there found. The amount of genes for which we failed to obtain a valid primer pair is represented by the left-most bar. Its highest value is observed for *Homo sapiens* reflecting above stated difficulty to find primer pairs without contained SNPs. We strive to decrease the number of yet missing PCR assays by increasing the number of input primer pairs to our algorithm. Within a transcript variant category we mostly find one variant that is amplified by the primers of the assay as is seen from Fig. 5 (bottom). However, the distribution of number of amplified variants has a long tail and there are assays that amplify more than 20 variants of the gene.

#### Comparison to other primer resources

There exist a variety of publicly accessible qPCR databases featuring a wide spread of supported species as well as total available assays, i.e. primer pairs.

The comparison to the most renowned databases, RTPrimerDB [15], GETPrime 2.0 [8], qPrimerDB [9], PrimerBank [5] and MrPrimerW [16] is summarised in Table 3.

**Table 3:** Comparison of PCRdrive to other public qPCR assay databases

		Number of species	Number of primer pairs	Batch download	Blast (cross-hybridisation)	SNP check	Secondary structure	Community workspace
qPCR databases	qPrimerDB	147	51,091,785	$\checked $	$\checked $	✕	$\checked $	✕
	GETPrime 2.0	13	1,175,874	$\checked $	$\checked $	$\checked $	✕	✕
	RTPrimerDB	21	7963	✕	$\checked $	$\checked $	$\checked $	✕
	PrimerBank	2	497,156	✕	$\checked $	✕	$\checked $	✕
	MRPrimerW	2	341,963,135	$\checked $	$\checked $	✕	✕	✕
	PCRDrive	3	283,226	✕	$\checked $	$\checked $	$\checked $	$\checked $

Our publicly available built-in primer design tool is based on the *primer3_core* and *blast* algorithms. It is thus comparable in its flexibility to renowned online tools as NCBI’s primer-blast [4]- but does not yet incorporate SNP check as does RexPrimer [17].

### User interface - PCRdrive

In this section we will explain how a user can access and retrieve information from our PCR assay database. The assay is integrated in our freely available and accessible online platform, PCRdrive. In “Online platform PCRdrive” section we will lay out the overall structure of the platform and explain some of the features that will help users take maximum advantage of the platform. In “Accessing the PCR assays” section we will explain in some detail how to successfully work with our assay database.

#### Online platform PCRdrive

**Fig. 6:**
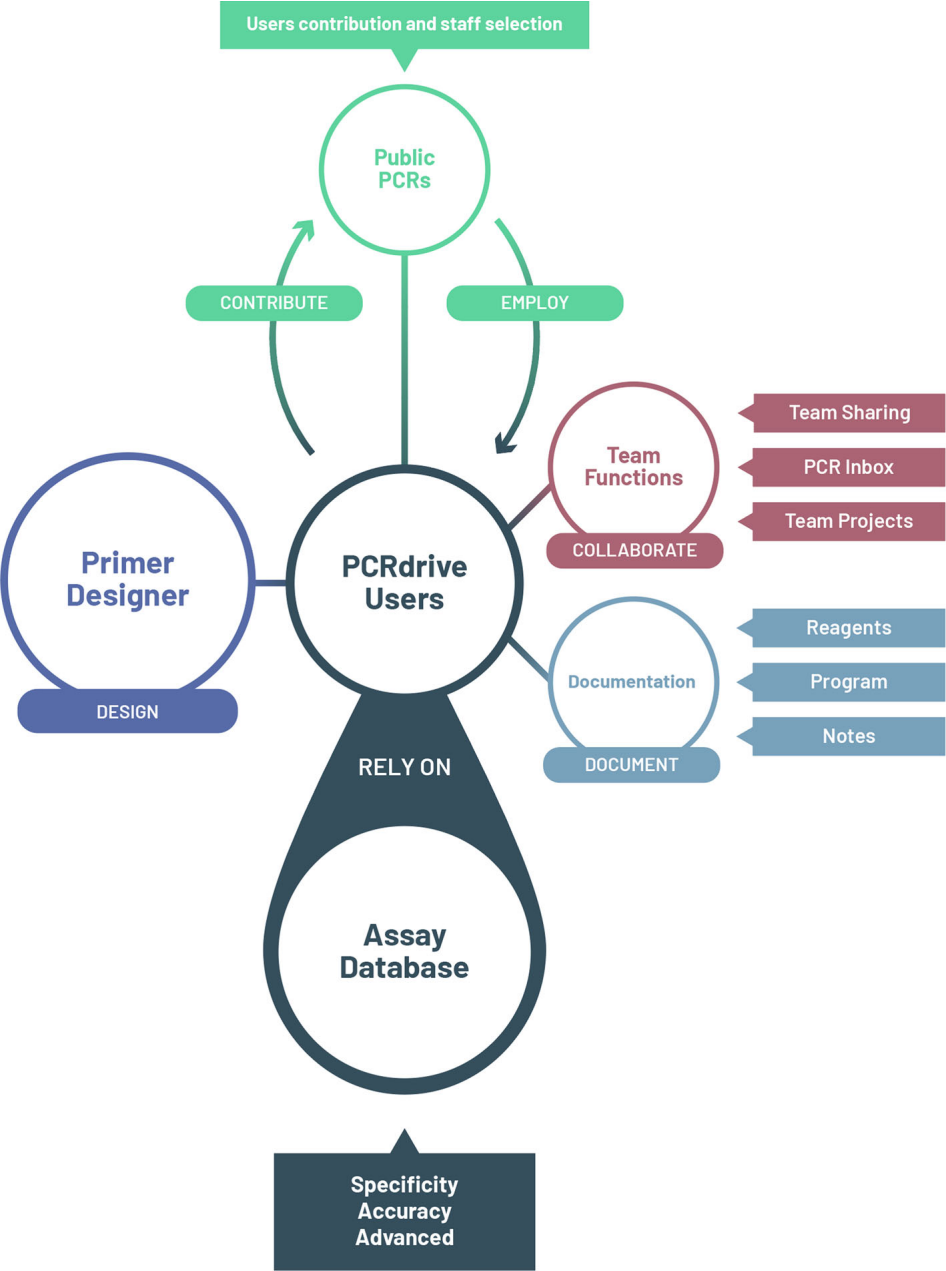
Overview of the PCRdrive platform

PCRdrive (https://pcrdrive.com) is a web based comprehensive, free platform for PCR users which includes a number of useful PCR-related workflow tools such as a primer designer with specificity control, documentation tools that aim at bringing together the international community of PCR users. It has been designed to cover all user needs arising within the design, conduct and report phases of PCR experiments. An overview of its features is given in Fig. 6. We have assembled useful information within wiki pages accessible under https://knowledge.pcrdrive.com/. Many questions new users might have regarding usage of the site are answered there.

The platform is secured by state-of-the-art cryptography methods (c. f. “PCRdrive” section) and privacy of input user data is strictly enforced. As a consequence a first-time user of PCRdrive thus has to complete a one-time registration process that will ask for the user’s valid email address as mandatory input. The validity of the email address is checked as part of the registration process. After successful registration a user may login to the platform and benefit from all its possibilities.

#### Accessing the PCR assays

The PCR assays database is accessible for the user from the PCRdrive dashboard or any other page from the left hand side menu by clicking the “Assay Database“ menu item (c. f. Fig. 7). The page will then load the most recent entries that have been added to the database. Each row provides the following information

1. the target ID,

**Fig. 7:**
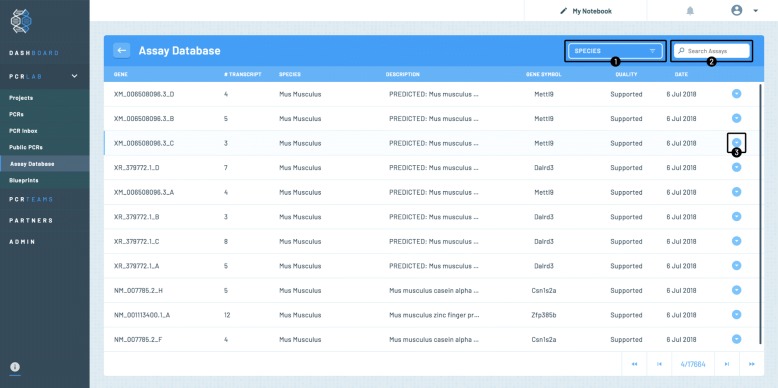
Screenshot showing the access to the PCR assay database. A typical listing of assays is shown for *Mus musculus*
2. the number of amplified gene variants,
3. the species,
4. the gene description,
5. the gene symbol,
6. a quality measure (currently only ’supported’ is shown for all assays but this field might contain further quality information about the assay in the future),
7. the date the assay was provided.

Assays that are marked with the yellow screw driver symbol are assays under current development that will be made available soon.

The list of currently available species may be obtained by clicking on the dropdown menu (labeled by ➀ in the figure) and the database may be filtered by one of the species listed when clicked upon. The user’s gene of interest may also searched for directly by entering the target ID into the search field (labeled by ➁ in the figure). In the bottom of the page, the user finds the number of pages of assays found given the current search criteria.

**Fig. 8:**
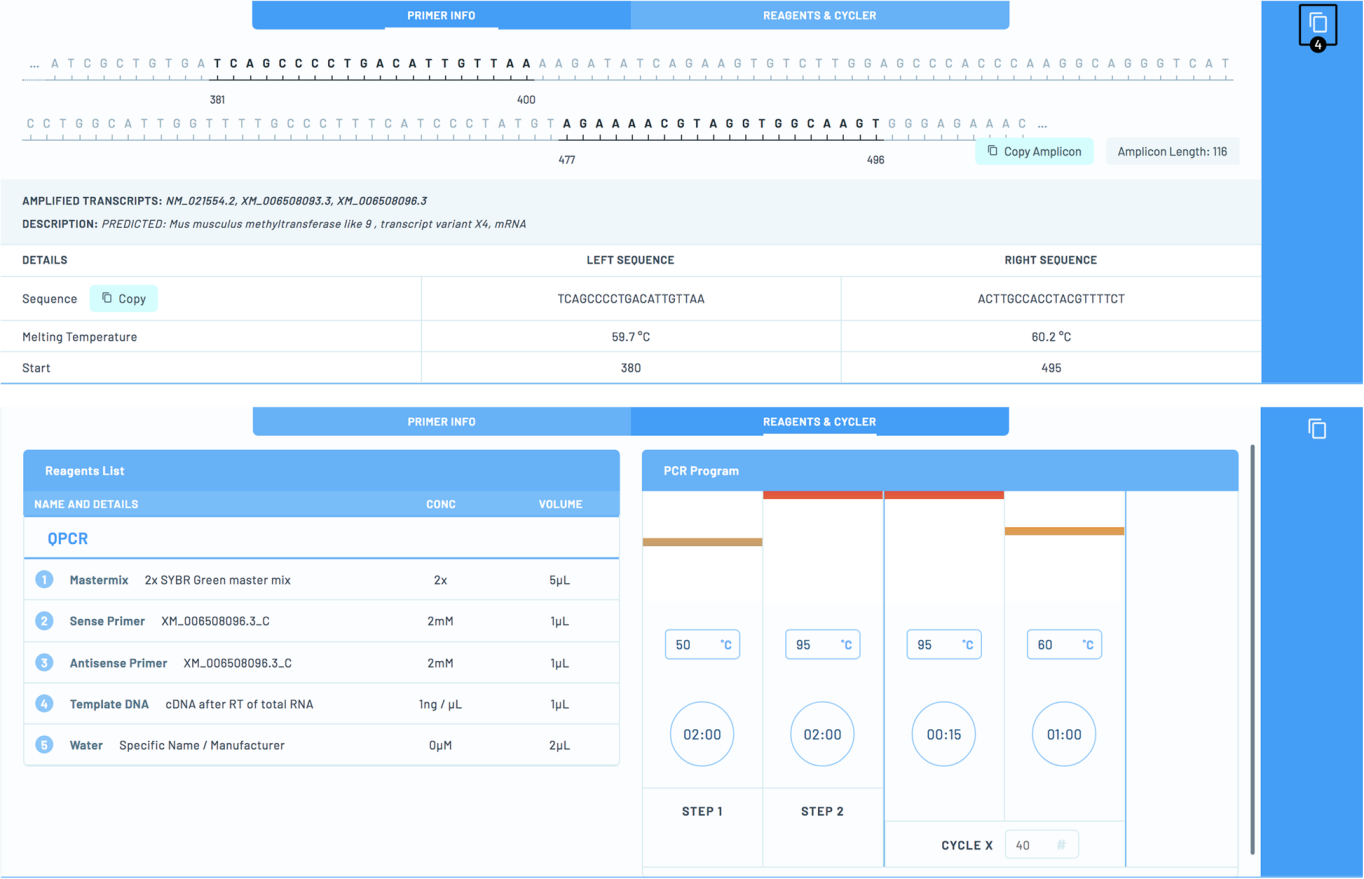
Screenshot showing details of a chosen assay about the primers used (top) and the optimal reagent and cycler protocol (bottom)

By clicking on an assay row or the arrow to the right (labeled ➂) details about the assay concerning the assay primers Fig. 8 (top) and concerning the reagent and cycler protocol to be used with the assay Fig. 8 (bottom) are displayed. The primer as well as the amplicon sequences may be copied to clipboard for further analysis. However the complete assay may also be copied to the user’s personal PCRs by clicking on the copy button (labeled by ➃ in the figure) where it may be documented and shared with team members or the whole PCR community (c. f. paragraphe Community features) etc.

#### Community features

PCRdrive was designed always having the community aspect in mind. The platform makes it easy for users to share his PCR work with others (be it a copied and adapted qPCR assay or a primer pair generated by the built-in primer designer), for which several opportunities exist.

The first of the sharing modes allows sharing PCRs within a team. To this end, a streamlined group sharing function allows to organize a team within PCRdrive (follow the ”PCR TEAM” link from the left-side menu). PCRdrive allows one to select a team administrator and then post or edit PCRs jointly. This private, collaborative space is an easy way to share each step of the process and trade information in a secure and organized group setting. Depending on the role within a team, members of the team may view or change team-shared work. The roles of team members are configurable as to adapt to laboratory staff changes.

Alternatively, results may be published as “Public PCRs” allowing other users to built on your work. Publicly shared PCRs can be easily copied by other users.

Third, it is possible to share a PCR directly with any other PCRdrive user using an inbox/outbox like mechanism known from regular email communication.

## Conclusion

While we are only reporting results for two species here, we will be constantly increasing the number of covered species. In the next round of updates, we will add the model species *Danio rerio* and *Drosophila melanogaster* to our database. We will then aim to include *Caenorhabditis elegans*, *Arabidopsis thaliana*, *Sus scrofa* as well as *Macaca fascicularis*.

As for the algorithm we intend to include analysis of secondary structure within the primer binding regions of the template in the upcoming versions.

## Availability and requirements

- Project name: PCRdrive
- Project home page: pcrdrive.com
- Operating system(s): Platform independent
- Programming language: C++, HTML5, JavaScript, Python
- License: Closed source
- Any restrictions to use by non-academics: None

## Abbreviations

ELN: Electronic lab notebook
MFE: Minimum free energy
PCR: Polymerase chain reaction
PLN: Paper lab notebooks
qPCR - Quantitative polymerase chain reaction; SNP: Single nucleotide polymorphism
Tm: Melting temperature
